# Development of a Preventive HIV Vaccine Requires Solving Inverse Problems Which Is Unattainable by Rational Vaccine Design

**DOI:** 10.3389/fimmu.2017.02009

**Published:** 2018-01-12

**Authors:** Marc H. V. Van Regenmortel

**Affiliations:** ^1^School of Biotechnology, CNRS, University of Strasbourg, Strasbourg, France

**Keywords:** antibody affinity maturation, bounded rationality, inverse problems, rational vaccine design, reductionism, reverse vaccinology, transmitted founder virus, virus inactivation

## Abstract

Hypotheses and theories are essential constituents of the scientific method. Many vaccinologists are unaware that the problems they try to solve are mostly inverse problems that consist in imagining what could bring about a desired outcome. An inverse problem starts with the result and tries to guess what are the multiple causes that could have produced it. Compared to the usual direct scientific problems that start with the causes and derive or calculate the results using deductive reasoning and known mechanisms, solving an inverse problem uses a less reliable inductive approach and requires the development of a theoretical model that may have different solutions or none at all. Unsuccessful attempts to solve inverse problems in HIV vaccinology by reductionist methods, systems biology and structure-based reverse vaccinology are described. The popular strategy known as rational vaccine design is unable to solve the multiple inverse problems faced by HIV vaccine developers. The term “rational” is derived from “rational drug design” which uses the 3D structure of a biological target for designing molecules that will selectively bind to it and inhibit its biological activity. In vaccine design, however, the word “rational” simply means that the investigator is concentrating on parts of the system for which molecular information is available. The economist and Nobel laureate Herbert Simon introduced the concept of “bounded rationality” to explain why the complexity of the world economic system makes it impossible, for instance, to predict an event like the financial crash of 2007–2008. Humans always operate under unavoidable constraints such as insufficient information, a limited capacity to process huge amounts of data and a limited amount of time available to reach a decision. Such limitations always prevent us from achieving the complete understanding and optimization of a complex system that would be needed to achieve a truly rational design process. This is why the complexity of the human immune system prevents us from rationally designing an HIV vaccine by solving inverse problems.

“Science is the acceptance of what works and the rejection of what does not. That needs more courage than we might think.” (Jacob Bronowski)

“The average scientist unequipped with the powerful lenses of conceptual insight, is a nearsighted creature, who cheerfully attacks each difficulty in the hope that it will be the last.” (Gilbert Lewis)

## Introduction

Most scientists have heard of the scientific method but usually have rather vague ideas about what it might be. In his popular book, translated into 15 languages, entitled *What is this Thing Called Science?* Chalmers (1) knew better than to formulate a philosophically articulated and rigorous account of a universal scientific method that would fully explain the success of science. It has also been argued convincingly that a universal scientific method applicable to all scientific disciplines is actually a myth (2) and there is, indeed, no scientific recipe book that scientists could use to make either discoveries (i.e., revealing something that existed all along but was unknown to anybody) or inventions (i.e., achieving something new that was not previously feasible, for instance, successfully vaccinating people against HIV infection). Furthermore, it is no longer considered feasible to simply derive a desirable invention such as an effective HIV vaccine from prior discoveries obtained by curiosity-driven basic research in immunology (3, 4).

The scientific method is often misrepresented as an invariable sequence of steps going from observations to hypothesis formulation, theory building and experimental verification rather than as a highly variable, complex and creative process (5). Attempts by philosophers of science to describe the methods used by scientists for obtaining new, reliable scientific knowledge often bear little resemblance to the way scientists behave in the laboratory. In their book entitled *Theories of Scientific Method* [(6), p. 2] Nola and Sankey quoted the physicist Richard Feynman who said: “Philosophy of science is about as useful to scientists as ornithology is to birds,” as well as the philosopher of science Imre Lakatos who quipped: “Most scientists tend to understand little more about science than fish about hydrodynamics.”

A major difficulty for explaining the success of the scientific method is due to the fact that scientific experiments in biology, for instance, produce results that regularly require continual self-correction because the data that are obtained are always approximations consisting of mixtures of “real” meaningful signals (relevant to what is being investigated) together with spurious “noise” due to random errors (7). This prevents absolutely certain and reliable knowledge to be obtained and explains why the scientific method has frequently failed to solve problems in the past. A satisfactory purely logical description of the scientific method should therefore be able to explain how the same method can either succeed or fail, a feat that has not yet been possible (1) [(2), p. 51].

The present review will discuss a number of invalid assumptions that have been detrimental to the search for a preventive HIV vaccine. Although several scientific journals are entirely devoted to the different approaches that can be used to solve inverse problems (*Journal of Inverse and Ill-posed Problems, Inverse Problems in Science and Engineering, Inverse Problems and Imaging*) many vaccinologists are unaware that the problems they are trying to solve are mostly inverse problems which consist in imagining what could bring about a desired outcome, for instance immunological protection against virus infection (8, 9). Investigators may not clearly differentiate between antigens and immunogens and often think of viral antigens as immunogens, implying that viral antigens and epitopes are able to trigger and generate immune responses although it is obvious that it is the immune system (IS) that produces antibodies (10).

This review will describe the reductionist mindset (11, 12), which has dominated molecular biology for half a century as well as a number of research paradigms that have been counterproductive for developing an HIV vaccine (13–15). It appears that the widespread expectation that the rational design of an HIV vaccine is likely to be more successful than tentative, small scale trial-and-error vaccine experimentation may have made it more difficult for vaccinologists to solve the inverse problems they are faced with.

## What are Inverse Problems?

Solving an inverse problem consists in first proposing a theoretical model for explaining, for instance, the absence of deleterious HIV infection in elite controllers, and subsequently demonstrating that by adjusting experimentally certain parameters in human ISs which the model predicts are crucial, it is indeed possible to obtain the desired outcome. Solving inverse problems therefore consists in identifying what are the causes that produced an observed effect, which requires reasoning by induction from a particular instance to a tentative generalization. Solving a direct problem, on the other hand, involves reasoning by deduction from a reliable premise or from an accepted physicochemical law to a particular instance, a procedure that in principle leads to the correct solution (16).

Many scientific problems in biology are direct, forward or downstream problems that can be solved by experimentally determining what are the effects that follow certain causes. Usually this involves elucidating particular causal mechanisms that are responsible for the occurrence of certain biological phenomena. A specific mechanism consists in interactions between the numerous parts of a complex biological system that are linked by direct causal relations and make it possible to systematically predict a result by providing an explanation for the occurrence of a given phenomenon (17). Solving a direct problem, therefore, always starts with known causes and makes the investigator observe, analyze, or calculate the results (18).

In physics and astronomy only a small number of physical forces exist that cause observable phenomena. For instance, the laws of universal gravitation together with certain initial conditions suffice to predict the occurrence of an eclipse because there are no external interferences in an interstellar vacuum.

The situation is completely different in the biological sciences because there are no universal laws in biology (19). No single event in biology can be said to be *the* cause of another event because it was necessary and sufficient on its own for the effect to occur [(20), p. 969]. Since any observed effect in complex biological systems always results from a network of causal interactions and internal regulations, an analysis in terms of a single causal factor instead of a multitude of contributory causes is never satisfactory (21).

Solving an inverse or upstream problem in biology requires inferring from a set of observations what are the multiple causes that produced certain effects. Medical diagnosis, for instance, is a typical inverse problem which consists in guessing the causes of a disease from some of its symptoms (22). An inverse problem thus starts with a result and requires that the investigator must try to imagine what could be the causes that produced it. This means that it is always necessary to first conceptualize a theoretical model that would account for what has been observed and subsequently to demonstrate experimentally that what the model predicts actually occurs. This is based on less reliable inductive reasoning which starts with the observation of particular instances in order to derive a general principle or model that unavoidably will only have a certain probability of being correct (18). Contrary to the solution of a direct problem which is easily obtained by deductive reasoning from correct premises or from known mechanisms or laws, solving an inverse problem is usually extremely difficult and may often be impossible.

In view of the complexity of the IS, a multiplicity of unknown causes may be responsible for what is observed when a patient has been infected or when a vaccine has been administered, and the inverse problem of developing an HIV vaccine could therefore have numerous solutions or possibly none at all. HIV vaccinologists, for instance, need first to conceptualize and articulate what are the multiple causes that sometimes allow the IS in a small number of individuals to elicit immune responses that protect against HIV infection. Subsequently, they need to make these rare causal chains occur regularly in large populations of genetically and physiologically heterogeneous human vaccinees. If the desired result is not achieved when the theoretical model is implemented, the inverse problem of developing a vaccine has not been solved!

Unfortunately, vaccinologists cannot follow the procedure followed by X-ray crystallographers when they derive the structure of a protein from the set of concentric rings or parallel bands observed in the X-ray diffraction pattern of the protein crystal. This procedure involves calculating by Fourier analysis the diffraction patterns produced by irradiating conceptual crystals with an imaginary beam of X-rays and choosing which of these theoretical diffraction patterns best matches the pattern observed with the protein of interest [(23), p. 98]. Such a procedure transforms the insoluble inverse problem posed by the uninterpretable diffraction pattern of the protein crystal into a series of hypothetical direct problems that are easily solved. Unfortunately, such an approach is not applicable for solving inverse problems in vaccinology.

## Can Systems Biology Help Solve Inverse Problems in Vaccinology?

Living organisms and biological systems are always organized into successive levels of increasing complexity from genes to RNAs, proteins, subcellular organelles, cells, tissues, and organs. There is no privileged level of causal determination at any of these levels since both bottom-up and top-down causal determination occur, in addition to horizontal causation operating within the boundaries of each of the different levels (24). There is, however, no single causal pathway that links gene sequence to protein sequence and conformation or to protein binding and function and which further extends to the upper levels of cells, tissues, organs and organisms (8). At each successive level of biological organization, innumerable interactions between genetic, epigenetic, biochemical, physiological and environmental factors occur and it is necessary to first study each level separately as an independent system (25) before the different levels can all be integrated to give rise to the notion of systems biology. Whereas top-down systems biology analyzes how higher levels of organization determine events at lower levels, bottom-up systems biology analyzes the functional properties, which emerge from a subsystem that has been characterized to a high level of mechanistic detail, in an attempt to predict its behavior and the occurrence of certain biological phenomena (26). Unfortunately, few kinetic parameters are currently measurable *in vitro* which severely limits the usefulness of the models that can be proposed at the different levels. Systems biology is therefore rarely capable of developing theoretical models that encompass numerous levels and could form the basis for investigating experimentally whether integrated immunological reactions associated with vaccine activity occur as predicted in models.

In an often-quoted review, Brenner (27) claimed that systems biologists are unable to solve inverse problems of physiology by deriving how a complex system works by simply observing its behavior, because a hypothetical model can never be more than one tentative, imperfect representation of reality. The human IS consists of an extremely intricate network of specialized cells, tissues and organs which is probably the most complex biological system that exists, with the exception of the human brain. In recent years, systems vaccinology which arose from systems biology, has employed high-throughput microarray technologies such as genomics, transcriptomics, proteomics, and metabolomics for describing the innumerable interactions that occur between all the parts of an IS in an effort to present a more holistic view of the entire system than is feasible when the constituents of each level are studied separately (28). This makes it possible, for instance, to identify gene signatures that may be able to predict the immunogenicity of certain vaccines, as well as the changes that occur in important cell subsets isolated from human blood, for instance CD4+ T cells, CD8+ T cells, and regulatory T cells. However, identifying signatures that predict vaccine efficacy does not necessarily provide a mechanistic insight for explaining how a particular vaccine stimulates protective immunity (29).

Transcriptional profiling of human immune responses to vaccination using peripheral blood mononuclear cells obtained from whole blood of vaccinees has demonstrated that immunization leads to up-or downregulation of many heterogeneous gene clusters, such as genes associated with innate immunity, dendritic cell activation, production of different cytokines, T cell receptor signaling, antibody titers, cell cycle progression, inflammatory responses, protein folding, mitochondrial dysfunction, etc. (30). Although some of these gene signatures are considered to be predictive of specific adaptive immune responses (31), the considerable measurement noise that is observed in the assays makes it difficult to distinguish correlations from causal relationships that may be linked to protection. Since a large number of up- or downregulated gene products act in combination to generate a biological function such as protection, it is extremely difficult to elaborate an integrated theoretical model that includes all the relevant causal factors and which could be tested in an attempt to solve a particular inverse problem. Furthermore, in non-linear dynamic systems, a complex network of internal regulations together with negative feedback and feed-forward control is always present, making it difficult to identify which causal factors are responsible for a biological effect (8, 24).

In his comprehensive overview of the promises of systems vaccinology for identifying effective vaccines, especially when correlates of protection are unknown, Pulendran (29) pointed out that several subsystems present in a completely integrated human IS are still insufficiently understood to provide the necessary knowledge required for developing a protective HIV vaccine. The inverse problem of solving each subsystem must first be achieved by developing appropriate theoretical models for each of them and showing experimentally that these models are correct. This is particularly difficult in the case of HIV because genetic, immunological and gut microbiome characteristic of the host must be considered together with the enormous antigenic variability of the virus. Since suitable theoretical models are not yet available for many of the IS subsystems, the problem of solving the more complex inverse problem arising in an integrated IS that incorporates all these subsystems is even more intractable. Such an holistic inverse problem can be called an ill-posed inverse problem which is defined as a problem for which insufficient information is available to make it possible to derive a plausible explanatory model that could be validated experimentally (32). Our ignorance of all the different parameters that control the IS subsystems does not allow us to elaborate a testable theoretical model that incorporates all the individual constituents and all the bottom-up and top-down causal links in each subsystem. It seems that several inverse problems will first have to be solved at the level of individual components of the IS before it would become possible to test the validity of an integrated theoretical model of immune protection against HIV infection.

This situation is somewhat reminiscent of the conundrum we face when trying to explain human consciousness (25). Consciousness is an emergent, systemic property and activity of the brain that cannot be attributed to a specific region of the brain but results when innumerable neuronal systems undergo many different processes. When we loose consciousness, following the administration of an anesthetic, we are unable to localize this phenomenon to a specific region of the brain, in the way sensory perceptions of vision or hearing originate in particular brain regions (33). Consciousness is thus an holistic and emergent brain property that results from the integration of a large number of individual brain processes. Similarly, the IS achieves protection against infection by an integrated combination of all its subsystems that cannot be explained by the activity of a single individual subsystem.

## Reductionist Thinking and Invalid Paradigms Have Hampered Attempts to Develop an HIV-1 Vaccine

Methodological reductionism has guided the development of molecular biology for half a century and was widely accepted because it was able to describe biological systems in terms of its physicochemical properties, giving rise to the belief that biology could be reduced to chemistry and physics (11, 12, 34).

Such a belief was consistent with the assertion of Crick (35): “The ultimate aim of the modern movement in biology is to explain all biology in terms of physics and chemistry.” When the double-helical structure of DNA was discovered in 1953, some commentators maintained that this discovery solved the mystery of life. Today, we recognize that the DNA structure only allowed us to understand in considerable detail, the molecular mechanisms involved in gene replication and expression and that it did not give us any insight on how genes actually lead to phenotypes.

For many years, biologists have had misgivings about the validity of reductionist explanations in biology (36, 37) because they were aware that biological systems possess so-called emergent properties that are not present in their constituent parts taken in isolation. Since dissecting the IS into its numerous components inevitably severs the connections that link the various parts together in a functionally integrated manner, essential features that regulate the IS are destroyed and it is no longer feasible to account for the behavior of the system as a whole (38).

The advent of Mabs revolutionized immunochemistry because it allowed immunologists to investigate the chemical and functional properties of polyclonal antibody responses by analyzing single epitope–paratope interactions. The use of Mabs, however, also promoted a reductionist approach because vaccinologists tended to focus on individual epitopes as elicitors of nAbs instead of studying larger antigenic surfaces that harbor a continuum of overlapping epitopes. They also neglected the fact that most protective immune responses are polyclonal and often involve synergistic effects arising from the collective neutralizing activities of Abs directed to different epitopes. Relying on a single epitope, identified by reverse vaccinology using one Mab for inducing a protective vaccine response is known to be less effective than using cocktails of several Env epitopes (39).

Reductionist thinking also made many vaccinologists accept that if an epitope of HIV Env binds to a broadly neutralizing (bn) Mab, this epitope should also be able to induce similar bnAbs in an immunized human host. The *chemical* nature of antigenicity was thereby confounded with the *biological* nature of immunogenicity and this led investigators to claim that they were designing a vaccine immunogen capable of generating protective antibodies whereas they were actually only improving the binding reactivity, i.e., the antigenicity, of a single HIV epitope (4). Another reductionist paradigm assumed that antibody protection against HIV infection could be analyzed at the level of individual epitope–paratope interactions although protection against infection is only meaningful in the biological context of particular cells and tissues and at the level of individual organisms. Additional methods for detecting emergent immunological patterns that are not detected by classical reductionist approaches have been described recently (40).

The concept of scientific paradigm was introduced by Kuhn (41) in his influential book *The Structure of Scientific Revolutions*. He argued that scientific investigators are always guided by assumptions and theoretical presuppositions, which he called paradigms, that determine the type of research and the experimental approaches they will use when trying to solve a particular problem. Since prevailing paradigms are usually not explicitly stated, investigators are often not aware of the tacit assumptions underlying their choice of paradigm and when they obtain results that are not consistent with these assumptions, they may fail to realize that their paradigm has been refuted and should be abandoned. This could then lead them to pursue unfruitful lines of investigation that may impede scientific progress. There is, indeed, evidence that in the field of HIV vaccine research several invalid paradigms have had a detrimental effect (15, 42). Paradigms are sometimes followed because they appear to have been useful for achieving earlier vaccine successes and they may then continue to be used even if they are incompatible with more recent immunological knowledge (4, 14). Other invalid paradigms used in HIV vaccinology have been discussed previously (4, 43–45).

## What is Structure-Based Reverse Vaccinology (SBRV)?

Two types of reverse vaccinology should be distinguished. Genome-based reverse vaccinology, introduced by Rino Rappuoli (46) predicts potential vaccine immunogens using bioinformatics analyses of entire bacterial genomes and has allowed the development of several effective bacterial vaccines (47). A completely different approach was proposed by Burton (48) for developing viral vaccines and it has been suggested that this should be called SBRV to clearly differentiate it from genome-based reverse vaccinology (49). Such a nomenclature is more explicit than calling the two procedures “reverse vaccinology 1” and “reverse vaccinology 2,” respectively (50).

Structure-based reverse vaccinology attempts to generate a vaccine by first determining the X-ray crystallographic structure of a complex between, for instance, an HIV Env and a bnMab, and then using the 3D structure of the Mab as a template to reconstruct the epitope by reverse engineering outside the context of the Env protein in which it was originally embedded. This approach was called vaccine design because it was assumed that the reconstructed epitope designed to fit the Mab might be able to acquire the immunogenic capacity to induce a polyclonal Ab response endowed with the same neutralizing capacity as the bnMab. However, what was being designed was an antigen able to bind the Mab and not a viral immunogen able to induce a protective immune response (45, 51).

The SBRV approach only focuses on the recognition process between a single epitope–paratope pair and it does not take into account that Abs are produced by the IS and not by the viral immunogen and that T cell help always play a crucial role. Properties of the IS such as the Ab gene repertoire, self-tolerance, the presence of appropriate helper and suppressor T cells, the production of cytokines, and various immunoregulatory mechanisms determine whether protective Abs will actually be elicited. Since SBRV investigators made no attempt to control these IS parameters, it is not astonishing that they did not succeed in developing an effective HIV vaccine (4, 10, 51–54).

Another reason for the lack of success of SBRV is that many investigators did not fully appreciate the extent of degeneracy of the IS. Degeneracy refers to the ability of many different Abs, B cell and T cell receptors to bind to the same ligand as well as to the ability of a single Ab or T cell receptor to bind many different antigens or peptides. This means that there is never a unique intrinsic epitope for any Ab molecule but always a diverse group of potential ligands (55, 56). The reason for this is that every combining site of an Ig, consisting of 50–70 residues distributed over six hypervariable loops, always harbors numerous, overlapping smaller paratope subsites of 10–20 residues that possess considerable conformational plasticity and flexibility, allowing them to bind a variety of epitopes (57, 58). Since each paratope represents only about 20–35% of the entire Ig combining site, the one epitope structure identified in an SBRV experiment does not necessarily correspond to the immunogenic structure that elicited the Mab used as template. When that Mab was present as a B cell receptor in the original immunized host, it could have been selected by other cross-reactive epitopes than by the one identified by X-ray crystallography in SBRV. Since an Ab is never monospecific for a single epitope, there is actually no reason to assume that the particular Mab used as template could only have been elicited by a single immunogenic structure, a fact that clearly invalidates the SBRV approach (45).

Another somewhat unexpected consequence of the polyspecificity of Abs is that the collective specificity of a polyclonal antiserum induced by a multiepitopic protein is always greater than the specificity of a single Mab raised against that protein. Since each Ab in a polyclonal antiserum is able to cross-react with a variety of different epitopes, these various cross-reactivities present in the antiserum are diluted out and will usually be masked. In contrast, there will be no such dilution effect in the case of a Mab and its ability to cross-react will therefore be observed more easily (59).

Although the crystallographic structure of many anti-HIV Env bnMabs has been elucidated in SBRV studies, it is evident that this knowledge has not made it possible to manipulate the IS so that it will elicit protective Abs. For instance, it has been known for many years that strongly neutralizing anti-HIV-1 Mabs often possess a very long heavy chain complementarity-determining region 3 (HCDR3) of 20–34 residues, compared to the average length of 16 residues of HCDR3 in human B cells (60). These long HCDR3 loops allow the Abs to penetrate the glycan shields of Env trimers and to reach highly conserved hydrophobic residues in the gp41 region of Env (61). It has also been established that long HCDR3s are not predominantly generated by somatic hypermutation-related insertions (62) but usually arise in humans during VDJ recombination and VH replacement events (63). As a result it is not possible to simply rely on stochastic Ab affinity maturation events for increasing the number of immune cells in a human IS that will contain long HCDR3s. One can only regret than humans ISs do not possess the large numbers of Abs with long CDR3s that are present in the IS of cows and allow these animals to produce large quantities of neutralizing anti-HIV bnAbs (64).

Most of the Env surface is covered with glycans, but when selective glycans are removed in the vicinity of the CD4 binding site, the underlying peptidic Env region becomes accessible to nAbs which can lead to an 1,000-fold increase in neutralization efficacy (65). Certain bnAbs of the VCR38 Ab lineage (66) that have HCRD3 loops of a normal length are able to bind neotopes at the V1/V2 apex of Env trimers. It has been known since the 1960s that the presence of identical subunits in viral capsids and spikes always gives rise to a quaternary structure and to new types of epitopes known as neotopes absent in the monomeric subunits (67, 68). Neotopes arise from the juxtaposition of residues from neighboring subunits or from conformational changes induced by intersubunit bonds. It is unfortunate that such neotopes capable of inducing neutralizing Abs were detected at the V1 V2 apex and other regions of Env only 30 years later. If neutralizing antineotope antibodies that do not bind to Env protomers had been detected earlier, it might have dissuaded investigators to try to use Env monomers as potential vaccine immunogens (69).

Epitope structures visualized by X-ray crystallography in Ab-antigen complexes usually provide little information on which exact structures of the immunogens are recognized by B cell receptors (BCRs) at the surface of lymphocytes. The exceptional plasticity, flexibility, and metastability of the HIV Env as well as the extensive Env conformational changes that occur during the transition from the prefusion to the postfusion state make it extremely difficult to know what structures are recognized by BCRs at different stages of the immunization process (70). Crystallographic analysis of the trimeric prefusion Env showed that V3 loop residues are not accessible to anti V3 Abs because they are occluded in a pocket formed by the V1V2 region that gives rise to neotopes. When Abs bind to such a neotope, a conformational change occurs which exposes the V3 loop to V3 Abs (71). Extensive conformational transitions are induced by the binding of Env Abs and these present considerable challenges for HIV vaccine development (4, 72).

After 15 years of unsuccessful attempts to develop an HIV vaccine by SBRV, this approach has lost its popularity with most HIV vaccinologists who no longer attempt to transform HIV epitopes of known structure directly into effective vaccine immunogens. However, the lure of a rational design approach for developing an HIV vaccine still exists as shown by the recent claim that “reverse vaccinology 2.0” (i.e., SBRV) shows great promise as a powerful vaccine design strategy (50).

## The Bottleneck of Stochastic Ab Affinity Maturation

Ab affinity maturation refers to a process whereby BCRs and Abs develop an increased affinity for binding to a particular antigen present in the light zone of germinal centers in secondary lymphoid tissue (73). When the local concentration of this initial viral antigen decreases over time, only BCRs that bind to it with the highest affinity may be triggered because the mass action law will allow such BCRs to recognize a partner present at a very low concentration. This process differs from what is observed when new bnAbs appear in the chronic phase of HIV infection, usually after a couple of years, when the virus has been integrated in the host genome. At that stage, intrahost evolutionary pressures lead to the appearance of many mutated viruses that are able to trigger new Abs recognizing novel viral antigens, a process that differs from what occurs when truly affinity matured Abs appear that react better with one particular viral antigen. The presence of increasing numbers of antigenic variants during chronic infection may induce the appearance of novel bnAbs that are not necessarily “affinity” matured since they recognize antigens that were not previously available. The capacity to neutralize the viral immunogen only becomes effective when Abs are subsequently produced by plasma cells and the selecting factor during BCR recognition may be the appearance at low concentrations of novel antigenic variants (74). This could explain why chronically HIV-1-infected humans from whom bnAbs are isolated do not benefit from the presence of such Abs for controlling virus replication (75, 76).

When it was demonstrated that HIV-1 epitopes recognized by affinity-matured bnMabs derived from infected individuals did not bind germline predecessors of these Abs (77, 78) it became evident that a lengthy process of Ab affinity maturation would be needed to obtain anti-HIV-1 Abs by vaccination. This meant that it would not be possible to directly engineer HIV epitopes by SBRV using germline-encoded Abs that had not undergone affinity maturation. The SBRV approach that could have worked with other viruses was doomed in the case of HIV because of the Ab affinity maturation bottleneck and of the enormous antigenic variability of the virus. This led many laboratories to initiate a major new research effort aimed at elucidating a huge number of possible stochastic maturation pathways that may lead from germline Abs to mature BCRs and to Abs endowed with a high mutation frequency (79–82). It is generally accepted today that a series of sequential immunizations with HIV epitopes that recognize increasingly mutated Abs will be necessary for obtaining a protective immune response in a genetically heterogeneous human population (83, 84), although the logistics of such an approach appear daunting. Such a vaccination strategy departs from the SBRV approach since it requires the unraveling of particular stochastic maturation pathways that need to be transferred to large numbers of genetically heterogeneous vaccinees. Numerous studies have attempted to identify which structural elements of Env recognized by different bnMabs should be present in the sequential immunogens needed for designing an HIV-1 vaccine (85–87) but it has not yet been possible to demonstrate empirically which structural features in immunogens are needed for eliciting a protective immune response.

Protein antigens and immunoglobulins are fairly flexible and dynamic molecules (88) and the plasticity of epitopes and paratopes has been compared with flexible keys interacting with adjustable locks (89). The X-ray crystallographic analysis of many Ab-antigen complexes has demonstrated that the binding process leads to important side-chain movements and changes in the backbone conformation of the two partners (90). The mutual adaptation and induced fit (91) that occur when the two partners interact often completely modifies the binding site structure originally present in the two free partners, with the result that the epitope structure observed in the complex may show little resemblance with the immunogenic structure that was recognized by the BCR during the immunization process. The recognition process between the epitope and a free Ab molecule may differ considerably from what occurs when this epitope binds to the cognate BCR embedded in a lipid membrane because hydrophobic interactions between membrane lipids and aromatic residues often lead to stronger binding (92). Many HIV Env epitopes are also chemically heterogeneous and may bind to Abs by involving specific peptidic interactions as well as numerous glycans or even lipids (4, 93).

## Can Rational HIV Vaccine Design Overcome the Limits of Bounded Rationality?

The term design refers to the intentional, deliberate conceiving of a novel object or process by an intelligent mind. The designer’s task is to pose and solve an inverse problem by imagining a theoretical model or process that would make it possible to obtain the desired outcome. It used to be believed that living organisms were designed by the preconceived plan of an intelligent deity although it is generally accepted today that organisms are shaped by the filter of Darwinian selection. There is, indeed, little anatomical evidence for the intentional design excellence of a mythical designer and, instead, plenty of evidence that utilitarian tinkering processes took place during evolution rather than design (94, 95). Design terminology also seems inappropriate for describing the activities of vaccine developers since investigators do not know how the IS succeeds in eliciting protective Abs and they need first to imagine a plausible theoretical model and subsequently to test its validity by demonstrating empirically that the predicted outcome actually does occur. Such a procedure hardly qualifies as intentional design and does not really justify the common belief that structure-based design is preferable to the empirical testing of HIV vaccine candidates. It may even seem odd that many authors still maintain that rational design is the best strategy for developing an HIV vaccine (96–100, 101) although they rarely made it clear what they mean by the term “rational.” Rational vaccine design is derived from the concept of “rational drug design” which uses the 3D structure of a biological target for designing molecules that will selectively bind to it and inhibit its biological activity. Such a computer-assisted approach based on molecular docking (102) is also feasible for improving the binding complementarity in an epitope–paratope pair by either designing an HIV Env epitope that binds better (103) or a paratope that binds better (104). In both cases, however, this amounts only to designing improved binders and not immunogens. As explained in the section “*What is structure-based reverse vaccinology*,” improving the binding capacity or antigenicity of an HIV Env epitope does not constitute rational vaccine design since an epitope that strongly binds a bnMab will not necessarily elicit Abs possessing the same neutralizing capacity as the bnMab. Abs are also often heterospecific, i.e., able to react better with other antigens than the one used in the immunization process that elicited the Ab; this demonstrates that antigenic and immunogenic properties are not necessarily located in the same regions of a protein molecule (59). In rational vaccine design, the term “rational” actually does not have the same meaning as in rational drug design that is based on molecular docking and it simply refers to the fact that the investigator is focusing on parts of the system for which molecular information is available.

The economist and Nobel laureate Herbert Simon introduced the concept of “bounded rationality” (105) to describe the intrinsic limitations of human cognition that are due to the many unavoidable constraints that always limit our ability to reach rational decisions. Such limitations are due, for instance, to insufficient and inaccurate information, a limited capacity to investigate any real-world complex system in all of its complexity and the limited time and resources that are available to humans. Since only a small number of possibilities can ever be investigated, humans cannot achieve the complete analysis and understanding of highly complex systems that they would require in order to make entirely rational decisions based on a complete knowledge of all relevant parameters. Instead of guaranteeing that correct solutions to a problem can be reached, bounded rationality forces humans to make decisions by following tentative heuristic procedures that cannot provide definitive solutions to inverse problems (106, 107). The reason for this is that all inductive and discovery procedures in science are heuristic principles that fail as algorithms in part because they do not represent logically valid argument forms (106). Multiple solutions to the same problem may also arise from the degeneracy of structure-function relationships present in biological systems which results from nearly equal, energetically available configurations. It is also not clear whether intrinsically stochastic elements can be incorporated into holistic paradigms in order to provide an understanding of the emergent properties characteristic of complex biological systems (108).

When all the relevant parameters of a complex system are actually not all known, which is the case, for instance, with the world economic system, totally rational choices and decisions are not feasible which explains why it was not possible to predict an event such as the world financial crisis of 2007–2008. Similarly, long term weather prediction also remains impossible as illustrated by the metaphoric “butterfly effect” which explains that small changes in initial conditions in a complex non-linear system lead to unpredictable consequences at a later stage. It is also impossible for vaccinologists to know in perfect detail the physiological and genetic backgrounds of all the ISs of human vaccines nor importantly their prior immunological exposure. The limitations imposed by bounded rationality does not allow them to predict the outcome of various immunological interventions, and this makes the strategy of rationally designing an HIV vaccine seem totally unrealistic. Only empirical evidence obtained by successfully manipulating and controlling an IS in order to achieve a certain degree of immune protection may allow investigators to reach plausible, but nevertheless tentative conclusions, on how best to proceed for attempting to develop an effective HIV vaccine. Since the aim of vaccinologists is to control immunological phenomena, they always need to interfere in the IS in order to gain knowledge about how to control it (109, 110). Increasing our knowledge of basic immunology and of HIV-1 antigenic structure is unlikely on its own, to allow us to rationally design an effective HIV-1 vaccine (4), although it may be useful for elaborating better theoretical models when trying to solve the numerous inverse problems that have until now impeded the development of an HIV vaccine.

## The use of Transmitted/Founder Virus as HIV Vaccine Immunogen

The preceding sections of this review may seem to some to be excessively negative and devoid of constructive suggestions about alternative approaches that could be investigated for developing an HIV vaccine. In this last section, it will be argued that most HIV vaccinologists have so far mainly studied the rather ineffective immune responses that occur after several years of chronic HIV infection. Since only a few studies have been devoted to the immune responses that occur during the initial acute phase of HIV infection, there clearly is a need to further investigate these responses that may be highly relevant for developing a protective vaccine.

Despite the high diversity of HIV populations found in infected individuals, it is remarkable that about 80% of heterosexually transmitted HIV-1 infections are caused by a single, so-called transmitted/founder (T/F) virus which is not the predominant variant in the donor. This phenomenon is known as the transmission bottelneck of HIV-1 (111). Compared to viruses present in chronic HIV infections, T/F viruses have about twice more Env spikes per virion, have shorter variable loops and fewer N-linked glycosylation sites, and are more efficiently captured by dendritic cells and transmitted to CD4+ T cells (112, 113). T/F viruses also utilize the chemokine receptor 5 as coreceptor and bind to an integrin receptor that transfers the virus from the genital tract to the gut-associated lymphoid tissue (GALT). Following infection by a single T/F virus, an acute phase of HIV infection occurs in the following month(s), which is characterized by the presence of infected CD4+ T cells in lymph nodes and intense viremia. Subsequently, the HIV proviral DNA is integrated in the host genome, seroconversion occurs as infected cells move from the GALT to the blood and chronic HIV-1 infection sets in, leading eventually to the progressive destruction of the host IS (114). In the chronic phase, Ab-based selection and virus escape occur continuously although this does not allow infected individuals to control virus replication (76). The bnAbs that appear during chronic infection 2–4 years after the initial infection are the ones that have been studied most extensively in SBRV experiments, although the initial immune response against T/F viruses, which also generates some neutralizing Abs, may be more relevant when trying to developing a preventive HIV-1 vaccine (115). It is somewhat paradoxical that most crystallographic studies of bnMabs were done with human anti HIV antibodies that are present after several years of chronic infection since these Abs are usually unable to control HIV infection in the infected individuals from whom they had been obtained.

Recently, the antigenicity and immunogenicity in guinea pigs of recombinant gp140 Env glycoproteins from T/F viruses and from chronic viruses have been compared in order to estimate their relative efficacy as potential vaccine immunogens (116). For this study, Env glycoproteins were selected that reacted with the largest number of available bnMabs, although a pronounced cross-reactive antigenicity may not necessarily entail a superior immunogenic efficacy against T/F viruses (59). The T/F Envs were found to induce Abs with the greatest neutralization breadth which suggests that T/F viruses may be suitable vaccine immunogens. The T/F Envs also induced Abs against the V3 loop.

In recent years, several vaccinologists have suggested that chemically inactivated HIV virions should be reconsidered as potential vaccine immunogens (114, 117, 118). Inactivated HIV vaccines had already been investigated more than 20 years earlier but these attempts failed because harsh and inappropriate inactivation methods were used. Recently, new and more reliable inactivation methods have been developed that no longer suffer from the defects of earlier methods (119). A particular attractive method consists in irreversibly destroying the infectivity of HIV by targeting the viral reverse transcriptase (RT) using UV irradiation of a photo-labeled RT inhibitor, a procedure that fully preserves the viral antigenic structure (114, 120). Such a targeted inactivation of RT could be used for inactivating various T/F viruses and could lead to the development of a whole-virus polyvalent vaccine that would possess double the amount of viral Env spikes per virion. Such a vaccine may possibly trigger an immune response in the acute infection phase and since the RT is inactivated, the vaccine may be able to prevent HIV integration and subsequent chronic infection (114). It is the neutralization of T/F viruses that are present initially and easily spread from cell to cell that may be the most relevant for preventing rapid viral dissemination. Improving the immunogenic properties of T/F viruses may thus be more important for developing a preventive HIV-1 vaccine than concentrating on the immunogens that give rise to bnAbs during the chronic phase of viral infection (121).

## Conclusion

It is often claimed that the IS is unable to control HIV infection because the virus continuously evolves new strategies and mechanisms in order to evade IS defenses. Such metaphoric language seems to attribute to HIV a goal directed capacity to defeat the host IS, although it is only the highly error-prone activity of the viral RT that is responsible for the enormous structural plasticity and antigenic variability of the Env glycoprotein, allowing the virus to evade immune control (71, 122). Since nAbs appear only very slowly during chronic infection, the Ab response lags behind the rapidly diversifying virus and the IS is unable to control HIV infection. Numerous additional, unsolved challenges have further contributed to our inability to control HIV-1 infection by vaccination (84). It also seems unrealistic to expect that passive immunization could provide the enormous quantities of recombinant Abs that would be needed to combat a world-wide pandemic. In spite of an immense, high caliber research effort backed by very considerable funding over several decades, it seems increasingly likely that the multiplicity of unsolved inverse problems that defy a rational vaccine design approach may prevent us from developing an effective HIV-1 vaccine. Although the time-honored vaccine approach of using chemically inactivated virions should urgently be tested with T/F viruses, we might have to accept that the HIV pandemic will not be brought under control by what would have been the ideal solution: an effective, preventive HIV-1 vaccine (123).

## Author Contributions

The author confirms being the sole contributor of this work and approved it for publication.

## Conflict of Interest Statement

The author declares that the research was conducted in the absence of any commercial or financial relationships that could be construed as a potential conflict of interest.
